# Dataset of multi-focus (Z-stack) images derived from liquid-based cervical cancer cytology specimens

**DOI:** 10.12688/f1000research.179164.1

**Published:** 2026-04-10

**Authors:** Takafumi Onishi, Tomoyuki Miyamoto, Yukihiko Osawa, Kazuki Shibahara, Makoto Nishimori, Hiromasa Yakushiji, Setsuyo Ohno, Eiji Ohno

**Affiliations:** 1 Department of Medical Technology and Sciences, Faculty of Health Sciences, Kyoto Tachibana University, Kyoto, Kyoto, Japan; 2 Research Center for Life and Health Sciences, KyotoTachibana University, Kyoto, Kyoto, Japan; 3 Department of Medical Life Sciences, School of Medical Life Sciences, Kyushu University of Medical Science, Nobeoka, Miyazaki, Japan; 4 Cancer Cell Institute, Kyushu University of Medical Science, Nobeoka, Miyazaki, Japan

**Keywords:** Multi-focus imaging; Z-stack; Cervical cancer; Liquid-based cytology; Deep learning; Pap smear; cytology; cytopathology.

## Abstract

**Background:**

In cervical cancer screening, cytotechnologists and cytopathologists integrate three-dimensional information by continuously adjusting the microscope’s focus to evaluate chromatin structure and nuclear morphology. However, most existing public datasets consist of single-focus 2D images, which do not fully reflect this clinical diagnostic workflow. This study presents the Cervical Cancer Cell Image Database: Multi-focus Cytology Dataset (CCCID) to bridge this gap.

**Methods:**

Cervical specimens were processed using the BD SurePath™ LBC technique and Papanicolaou staining. Digitization was performed using a NanoZoomer-XR scanner. For 639 unique fields of view (FOVs), a Z-stack consisting of 11 focal planes was captured at 1.0 μm intervals, resulting in 7,029 images (384 × 384 pixels). Ground-truth labels were established only when six board-certified expert cytotechnologists reached 100% consensus.

**Conclusions:**

The CCCID provides a high-reliability benchmark for developing machine-learning models that utilize axial (Z-axis) information. It is highly valuable for advancing three-dimensional nuclear morphology analysis, cell segmentation in overlapping clusters, and the evaluation of focus-fusion algorithms in digital cytopathology.

## Introduction

Cervical cancer remains a leading cause of cancer-related deaths among women globally, and cytology is central to its early detection. ^1^ Liquid-based cytology (LBC) is a widely used standard method because of its specimen uniformity. Although image analysis using deep learning has flourished, existing public datasets such as the Herlev dataset, ^2^ Cervix93, ^3^ SIPaKMeD, ^4^ and CRIC Cervix ^5^ primarily consist of single-focus 2D static images. These do not fully reflect the actual diagnostic process in which cytotechnologists and pathologists integrate three-dimensional information by continuously adjusting the focus to evaluate chromatin structure, nuclear membrane irregularities, and cell overlapping. ^6^ Even with LBC, cells and nuclei retain a thickness of several to over 10 micrometers. Single-plane 2D images risk losing critical information, such as 3D nuclear morphology. Therefore, this dataset was constructed to provide multi-focus image sequences (Z-stacks) of cervical LBC specimens. By including continuous focal depths for each field of view, this dataset enables the development of analytical methods and artificial intelligence models that consider 3D morphological information, thus reflecting conditions closer to real-world clinical practice.

## Materials and methods

1. Specimen Preparation and LBC Processing
  Cervical cytology specimens were collected from patients at Nobeoka Prefectural Hospital and Kawasaki Medical University. All samples were processed using the BD SurePath™ LBC system (BD Diagnostics, Burlington, NC, USA), which employs a density gradient enrichment process to provide a representative monolayer of cells. The processed slides were stained using the standard Papanicolaou staining method to visualize nuclear and cytoplasmic features. This study was conducted in accordance with the Declaration of Helsinki and was approved by the Institutional Review Boards of all participating institutions, including the Ethics Committee of Kyushu University of Medical Science (Approval No. 17–19). Informed consent was obtained using an opt-out procedure approved by the ethics committees. The study included only adult participants, and all data were anonymized prior to image extraction and annotation. Written informed consent was waived in accordance with the opt-out procedure approved by the ethics committees, given the retrospective nature of the study and the use of fully anonymized samples.
2. Whole-slide imaging and Digital Acquisition
  The prepared LBC slides were digitized using a NanoZoomer-XR whole slide imaging scanner (Hamamatsu Photonics, Shizuoka, Japan). Scanning was performed using a 20× objective lens. The proprietary software of the scanner was used to generate whole-slide images in the .ndpi format.
3. Fields of view (FOVs) Selection and Multi-focus (Z-stack) Extraction
  Specific FOVs representing typical cytological features of each Bethesda category were identified from whole-slide images. For each selected FOV, a multi-focus image sequence (Z-stack) was generated. The extraction process involved capturing 11 distinct focal planes with a vertical interval of 1.0 μm between each plane. The focal range was centered on the optimal focus determined by the expert system, covering a total depth of 10 μm (5 μm above and below the center). The extracted images were cropped into 384 × 384 pixel tiles and converted from the raw.ndpi format to the JPG format with a resolution of 96 dpi for standardized use in machine-learning workflows.
4. Annotation and Consensus Validation
  The annotation of the dataset was conducted by six board-certified expert cytotechnologists. To facilitate the annotation process, a custom software, “Annotation Image Creation Tool,” developed by Proassist Ltd., (Osaka, Japan) was used. The annotation protocol was as follows. Each expert independently reviewed the multi-focus image sequences for each FOV. The FOVs were classified into one of the following four categories based on the Bethesda System: negative for intraepithelial lesion or malignancy (NILM), low-grade squamous intraepithelial lesion (LSIL), high-grade squamous intraepithelial lesion (HSIL), squamous cell carcinoma (SCC). In this dataset, these are categorized as “SCC_etc,” which includes both SCC and adenocarcinoma (AC). A strict consensus rule was applied for inclusion in the final dataset; an FOV was included only if all six experts reached a 100% agreement on the diagnostic classification. The FOVs that did not achieve unanimous agreement were excluded from the dataset to ensure the highest possible ground-truth reliability.
5. Final Dataset Organization
  The final validated dataset, consisting of 639 FOVs (7,029 total images), was organized into the directory structure described in the Data description section. Each file was named according to its diagnostic class, FOV index, and Z-stack index to facilitate automated processing.

### Data description

CCCID is organized into four main directories named according to the Bethesda System: “NILM,” “LSIL,” “HSIL,” and “SCC_etc” ( Table 1). Each directory contains subfolders corresponding to specific cellular or pathological types ( Table 2). The “NILM” directory contains nine subfolders representing normal or benign cellular components: 1. Superficial-Intermediate Cells, 2. Parabasal-Basal Cells, 3. Glandular Cells (Isolated), 4. Glandular Cells (Cluster), 5. Squamous Metaplastic Cells, 6. Repair Cells, 7. Atrophic Vaginitis, 8. Macrophages, and 9. Neutrophils. The “LSIL” directory contains four subfolders: 10. Superficial-type Dysplastic Cells, 11. Intermediate-type Dysplastic Cells, 12. Superficial-type Koilocyte, and 13. Intermediate-type Koilocyte. The “HSIL” directory contains two subfolders: 14. Deep-layer Dysplastic Cells and 15. Carcinoma in situ Cells. The “SCC_etc” directory contains two subfolders: 16. Squamous Cell Carcinoma and 17. Adenocarcinoma.

**Table 1.** Dataset composition of the cervical cancer cell image database: Multi-focus cytology dataset.

Category	Folder name	No. of FOVs	Images per FOV	Total JPG files
Negative for intraepithelial lesion or malignancy	NILM	273	11	3,003
Low-grade squamous intraepithelial lesion	LSIL	93	11	1,023
High-grade squamous intraepithelial lesion	HSIL	69	11	759
Squamous cell carcinoma and Adenocarcinoma	SCC_etc	204	11	2,244
Total		639		7,029

Fields of view (FOVs), negative for intraepithelial lesion or malignancy (NILM), low-grade squamous intraepithelial lesion (LSIL), high-grade squamous intraepithelial lesion (HSIL), squamous cell carcinoma (SCC_etc).

**Table 2.** Detailed cellular composition of the cervical cancer cell image database: Multi-focus cytology dataset.

Main folder name	Subfolder name (cell type)	No. of FOVs	Images per FOV	Total JPG files
NILM	1. Superficial-Intermediate Cells	30	11	330
	2. Parabasal-Basal Cells	30	11	330
	3. Glandular Cells (Isolated)	30	11	330
	4. Glandular Cells (Cluster)	13	11	143
	5. Squamous Metaplastic Cells	21	11	231
	6. Repair Cells	6	11	66
	7. Atrophic Vaginitis	21	11	231
	8. Macrophages	91	11	1,001
	9. Neutrophils	31	11	341
LSIL	10. Superficial-type Dysplastic Cells	11	11	121
	11. Intermediate-type Dysplastic Cells	29	11	319
	12. Superficial-type Koilocyte	32	11	352
	13. Intermediate-type Koilocyte	21	11	231
HSIL	14. Deep-layer Dysplastic Cells	11	11	121
	15. Carcinoma In Situ Cells	58	11	638
SCC_etc	16. Squamous Cell Carcinoma	175	11	1,925
	17. Adenocarcinoma	29	11	319
	Total	639		7,029

Fields of view (FOVs), negative for intraepithelial lesion or malignancy (NILM), low-grade squamous intraepithelial lesion (LSIL), high-grade squamous intraepithelial lesion (HSIL), squamous cell carcinoma (SCC_etc).

Each subfolder contains individual image files in JPG format, representing specific FOVs and their corresponding multi-focus planes.

The image files follow the naming convention: [Serial Number]_(CenterX, CenterY, Z-stack Index).jpg.

• [Serial Number]: A unique sequential identifier assigned to each captured FOV.
• [Center X, Center Y]: X and Y coordinates of the center of the FOV within the specimen (μm).
• [Z-stack Index]: The focal plane number, ranging from 0 to 10 (representing 11 layers).

For example, the file “001_(3059,11012,0).jpg” represents the first captured image, located at specimen coordinates (3059 μm, 11012 μm), with a focal plane index of 0. The files “001_(3059,11012,0).jpg” through “001_(3059,11012,10).jpg” constitute the complete 11-layer Z-stack for the first FOV.

**Figure 1.**
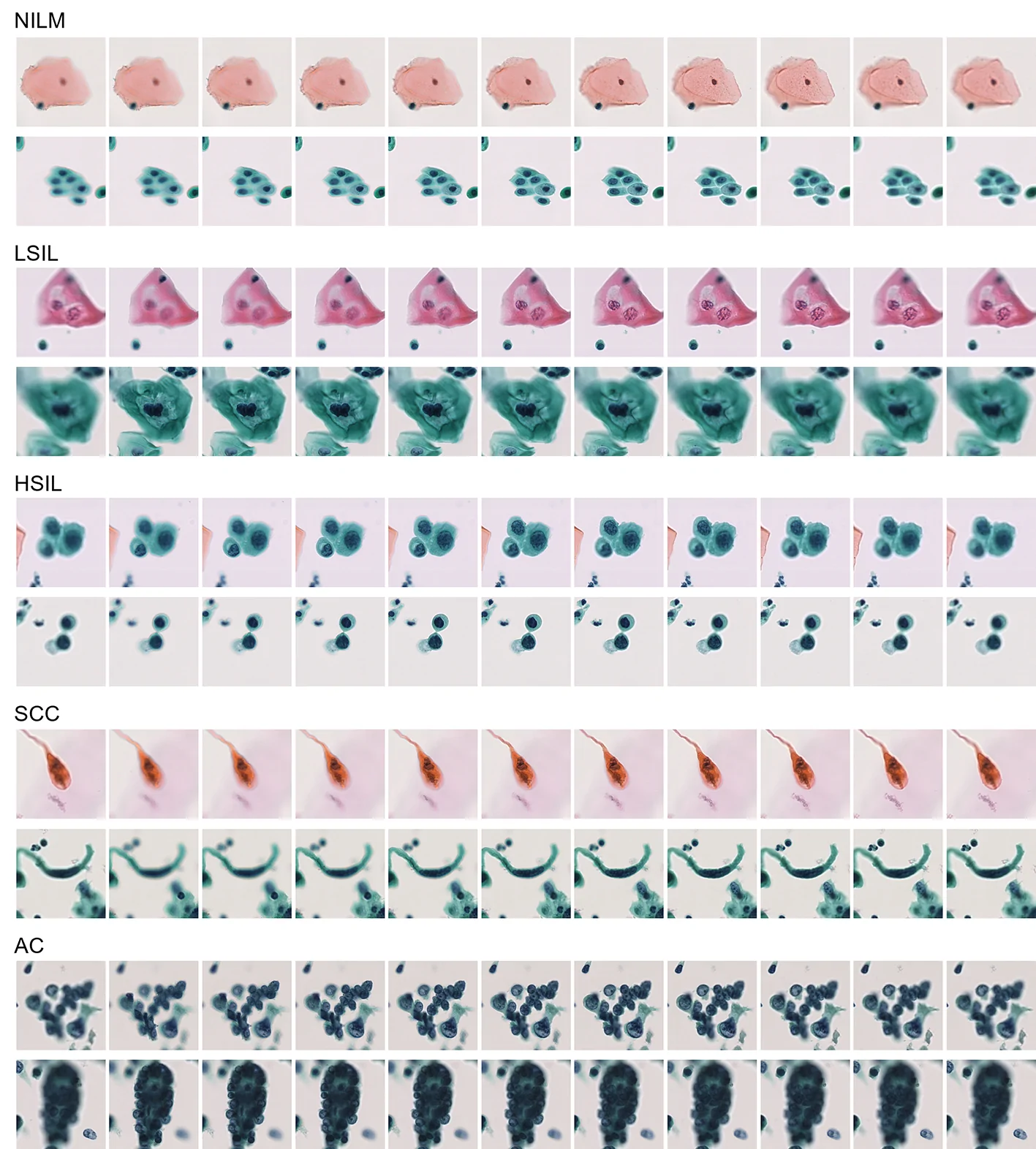
The representative multi-focus (Z-stack) images for each category contained in the Cervical Cancer Cell Image Database: Multi-focus Cytology Dataset. Negative for intraepithelial lesion or malignancy (NILM), low-grade squamous intraepithelial lesion (LSIL), high-grade squamous intraepithelial lesion (HSIL), squamous cell carcinoma (SCC), and adenocarcinoma (AC).

All images are 384 × 384 pixels with a resolution of 96 dpi. The Z-stack index 0 to 10 corresponds to a focal range captured at 1 μm intervals, providing a comprehensive volumetric view of the cellular and nuclear morphology. A representative image is shown in Figure 1.

### Value of the data

CCCID provides unique multi-focus (Z-stack) image sequences of cervical cytology, addressing the limitations of existing single-focus 2D datasets by reflecting the actual diagnostic process used by cytotechnologists and cytopathologists. These data are valuable for developing and evaluating deep learning models that require three-dimensional morphological information, such as chromatin distribution and nuclear membrane irregularities, which are often blurred or lost in single-plane images. The dataset features high ground-truth reliability, as every included image was validated through 100% consensus among six expert cytotechnologists, minimizing interobserver variability in the training data. Researchers can reuse this dataset to benchmark computer-aided diagnosis systems, specifically for testing algorithms designed to handle overlapping cells or thick cell clusters common in LBC specimens. The inclusion of 11 focus layers at 1 μm intervals allows for the exploration of focus-fusion (Extended Depth of Field) algorithms and the study of how vertical focal shifts impact the accuracy of automated cell classification.

### Limitations

Although CCCID provides a high-quality multi-focus resource, several limitations should be noted. First, the data were collected using a specific Whole Slide Imaging scanner (NanoZoomer-XR) and a single LBC preparation method (BD SurePath™). Therefore, the visual characteristics of the images may differ from those produced by other scanners or preparation techniques, such as ThinPrep. Second, the dataset focuses on typical diagnostic images where six expert cytotechnologists reached a 100% consensus. This means that highly atypical or “borderline” cases, which often cause diagnostic disagreement in clinical practice, were intentionally excluded. Third, the number of FOVs varies across categories, resulting in class imbalance among the different cell types. Finally, the Z-stack range is fixed at 11 layers with 1 μm intervals.

## Ethical considerations

In all cases, informed consent was obtained from the patients using an opt-out procedure. This study was conducted in accordance with the Declaration of Helsinki and was approved by the Institutional Review Boards of all participating institutions, including the Ethics Committee of Kyushu University of Medical Science (Approval No. 17–19). All patient data were fully anonymized prior to the image extraction and annotation process to ensure the protection of personal information.

## Consent to publish

Cytology samples were used in anonymized form. Consent for the use of clinical samples and associated images for research and publication was obtained through an opt-out procedure approved by the institutional ethics committees, including the Ethics Committee of Kyushu University of Medical Science (Approval No. 17–19).

## Data Availability

Zenodo: Cervical Cancer Cell Image Database: Multi-focus Cytology Dataset (CCCID). https://doi.org/10.5281/zenodo.18904734. ^7^ This project contains the following underlying data:
•
CCCID_NILM_part1.zip.7z This file contains image data for: 1. Superficial-Intermediate Cells, 2. Parabasal-Basal Cells, 3. Glandular Cells (Isolated), and 4. Glandular Cells (Cluster). •
CCCID_NILM_part2.zip.7z This file contains image data for: 5. Squamous Metaplastic Cells, 6. Repair Cells, 7. Atrophic Vaginitis, 8. Macrophages, and 9. Neutrophils. • CCCID_LSIL.zip.7z This file contains image data for: 10. Superficial-type Dysplastic Cells, 11. Intermediate-type Dysplastic Cells, 12. Superficial-type Koilocyte, and 13. Intermediate-type Koilocyte. • CCCID_HSIL.zip.7z This file contains image data for: 14. Deep-layer Dysplastic Cells and 15. Carcinoma in situ Cells. •
CCCID_SCC_etc.zip.7z This file contains image data for: 16. Squamous Cell Carcinoma and 17. Adenocarcinoma. Data are available under the terms of the Creative Commons Attribution 4.0 International license (CC-BY 4.0).
