# Supplementary material for: Dataset of multi-focus (Z-stack) images derived from liquid-based cervical cancer cytology specimens
Source: F1000Res. 2026 Apr 10;15:502. [Version 1] doi: 10.12688/f1000research.179164.1 (PMC13560679; doi:10.12688/f1000research.179164.1)
Supplement: Peer review history

# Reviewer report: Reviewer response for version 1

**DOI:** 10.5256/f1000research.197645.r503339

**Published:** 2026-08-08

**Authors:** Nisreen Albzour

**Affiliations:** 1 Binghamton University, Binghamton, USA

This Data Note presents a well-organized and valuable multi-focus cervical cytology dataset. The methodology and annotation process are clearly described. I recommend clarifying the number of unique patients/specimens and whether multiple FOVs originate from the same specimen, as this information is important for preventing data leakage in machine-learning train/test splits.

Are sufficient details of methods and materials provided to allow replication by others?

Yes

Is the rationale for creating the dataset(s) clearly described?

Yes

Are the datasets clearly presented in a useable and accessible format?

Yes

Are the protocols appropriate and is the work technically sound?

Yes

Reviewer Expertise:

Machine learning and deep learning in medical imaging, cervical cancer classification, and reliability evaluation.

I confirm that I have read this submission and believe that I have an appropriate level of expertise to confirm that it is of an acceptable scientific standard.

# Reviewer report: Reviewer response for version 1

**DOI:** 10.5256/f1000research.197645.r489794

**Published:** 2026-06-25

**Authors:** Vedrana Ivezic

**Affiliations:** 1 University of California Los Angeles, Los Angeles, California, USA

This paper presents a dataset of cervical cancer cytology multi-focus images. A current limitation of available public cytology datasets is that they contain only single focus 2d images. However in practice, cytologists evaluate multiple focal planes to diagnose a cytology specimen which allows them to resolve common challenges like stacked cells or blur. This dataset provides cytology z-stack images; enabling training and benchmarking of models more reflective of the standard clinical workflow. Each selected image is a Papanicolaou stained cervical specimen with 11 focal planes captured at 20x magnification. Bethesda labels were determined based on cytotechnologist review and required 100% agreement to remain in the final dataset. Data is available online on Zenodo and follows the structure outlined in the paper.

 Comments that need to be addressed:

- Many cytology datasets scan images at 40x magnification which follows clinical workflow (Pérez Bianchi et al. 2025, Hussain et al. 2020, Welch et al. 2024). Further clarification of why 20x rather than 40x magnification is necessary to position this dataset among the other publicly available cytology datasets.
- While only retaining specimens with 100% inter-rater agreement in the final dataset, could the authors include the additional samples along with the label distributions? Including this information is vital for developing models that handle more difficult samples (as noted in the limitations) and for building models with soft labels. Users of the dataset can simply exclude these samples if they only want the 100% agreement on label.
- Can the authors clarify why every image has the same number of focal planes and why 11 was chosen? Do some images ever need more than 11 planes to fully make out the present sample?

Are sufficient details of methods and materials provided to allow replication by others?

Yes

Is the rationale for creating the dataset(s) clearly described?

Yes

Are the datasets clearly presented in a useable and accessible format?

Yes

Are the protocols appropriate and is the work technically sound?

Yes

Reviewer Expertise:

Machine learning and deep learning applications in cytology imaging. Self-supervised learning.

I confirm that I have read this submission and believe that I have an appropriate level of expertise to confirm that it is of an acceptable scientific standard.

## References

[1] : Liquid based-cytology Pap smear dataset for automated multi-class diagnosis of pre-cancerous and cervical cancer lesions. *Data in Brief* .2020;30: 10.1016/j.dib.2020.105589 10.1016/j.dib.2020.105589 PMC718651932368601

[2] : RIVA: An Image Dataset of Conventional Pap Smear Cytology with Multiple Independent Annotations. *Scientific Data* .2025;12(1) : 10.1038/s41597-025-06280-2 10.1038/s41597-025-06280-2 41365892 PMC12727731

[3] : BMT: A Cross-Validated ThinPrep Pap Cervical Cytology Dataset for Machine Learning Model Training and Validation. *Scientific Data* .2024;11(1) : 10.1038/s41597-024-04328-3 10.1038/s41597-024-04328-3 39732723 PMC11682344
